# Clinically important estimates of improvement after septoplasty

**DOI:** 10.1017/S0022215123000993

**Published:** 2023-11

**Authors:** Rolf Haye, Liv Kari Døsen, Magnus TarAngen, Caryl Gay, Are Hugo Pripp, Olga Shiryaeva

**Affiliations:** 1 Department of Otorhinolaryngology, Lovisenberg Diakonale Hospital, Oslo, Norway; 2 Department of Otorhinolaryngology Head and Neck Surgery, Rikshospitalet, Oslo University Hospital, Oslo, Norway; 3 Department of Quality and Analysis, Lovisenberg Diakonale Hospital, Oslo, Norway; 4 Oslo Center of Biostatistics and Epidemiology, Reasearch Support Services, Oslo University Hospital, Oslo, Norway; 5 Department of Patient Safety and Research, Lovisenberg Diakonale Hospital, Oslo, Norway

**Keywords:** Patient outcome assessment, nasal surgical procedures, nasal obstruction, minimal clinically important difference, ROC curve

## Abstract

**Objective:**

A statistically significant improvement in nasal obstruction ratings following septoplasty is not necessarily clinically important. This study aimed to establish useful measures of septoplasty success, namely the minimal clinically important difference and the desirable clinically important difference.

**Methods:**

Patients rated nasal obstruction on a 0–100 visual analogue scale pre-operatively and at 5.5 months post-operatively. Global outcome rating (completely, much, or somewhat improved, unchanged or worse) served as the anchor post-operatively. Minimal clinically important difference is the visual analogue scale value between ‘somewhat improved’ and ‘unchanged’, and the desirable clinically important difference is that between ‘much’ and ‘somewhat improved’.

**Results:**

Statistically significant improvement in visual analogue scale scores was not clinically important. The minimal clinically important difference (daytime value of 9.5) represented 15.1 per cent improvement and the desirable clinically important difference (daytime value of 28.5) represented 45.2 per cent, without gender or age differences.

**Conclusion:**

Clinical success can be defined using a minimal clinically important difference of 15 per cent improvement over a patient's baseline value. Other studies’ ratings of ‘satisfactory’ outcome coincided with a desirable clinically important difference of 45 per cent over baseline. These values are suggested as relevant indicators of septoplasty success.

## Introduction

Outcomes of nasal septal surgery are often rated using subjective scales of nasal obstruction such as the Nasal Obstruction Symptom Evaluation (‘NOSE’) survey, the 22-item Sinonasal Outcome Test (SNOT-22) and visual analogue scales (VASs). The outcomes are often regarded as successful if the difference between pre- and post-operative ratings is found to be statistically significant. However, statistically significant improvements are not necessarily clinically important. Establishing levels of clinically important differences is therefore valuable, particularly when comparing outcomes across studies, surgical techniques and patient groups, and over time.

Some clinically defined outcomes in nasal septal surgery have already been published^1–7^ in studies using organ-specific instruments. However, they differ in their definition of what constitutes clinical success. Some studies have called for further investigations on clinically important improvements in septal surgery.^5^ We, therefore, wanted to relate the change in VAS ratings of nasal obstruction to subjective ratings of change in nasal obstruction using a five-point global scale of the surgical outcome ranging from ‘completely improved’ to ‘worse’, to establish the minimal clinically important difference. Secondarily, we wanted to establish an estimate that distinguishes between ‘much’ and ‘somewhat’ improved, which we would call a desirable clinically important difference in nasal obstruction following nasal septal surgery. Both the minimal clinically important difference and desirable clinically important difference might be used in evaluating the results of septal surgery and as guidelines for the quality control of septal surgery.

## Materials and methods

The study was approved by the Ethics Committee of Lovisenberg Diakonale Hospital. Patients who underwent septoplasty with or without turbinate surgery at Lovisenberg Diakonale Hospital from April 2014 to September 2019 were included. They were aged at least 17 years and did not have any other nasal or sinus disease except allergy.

We routinely use the Nasal Surgical Questionnaire^8^ in assessing the results of nasal septal surgery. The pre-operative version is completed in the morning on the day of surgery. The questionnaire contains separate VASs for nasal obstruction during the day and at night. Each VAS has a 10 cm line, with the left end representing no obstruction and the right end reflecting complete obstruction. The patients were asked to rate their sense of nasal obstruction on each of the scales with a vertical line. The score is measured in millimetres from the left-hand side of the scale.

The post-operative version of the Nasal Surgical Questionnaire has an added question about the retrospective sense of change in nasal obstruction following surgery: ‘Is your breathing now completely, much, or somewhat improved, unchanged or worse?’ These global ratings were assigned a status of 1 to 5: status 1 = completely improved, status 2 = much improved, status 3 = somewhat improved, status 4 = unchanged, and status 5 = worse. The patients were asked to respond to the items on the Nasal Surgical Questionnaire, indicating how they felt on a normal day without any infection. The Nasal Surgical Questionnaire was mailed to each patient five and a half months post-surgery, together with a cover letter signed by a surgeon at the department and a prepaid return envelope. Three weeks later, a reminder with the same questionnaire was mailed to those who had not returned the first questionnaire.

There are two commonly used methods to establish the minimal clinically important difference: anchor-based and distribution-based methods.^9–11^ As the US Food and Drug Administration^10^ has recommended the anchor-based method, we chose this as the primary method for establishing the minimal clinically important difference in this study. However, given that other studies on septoplasty outcomes^1–3^ have used the distribution-based method, for the purpose of comparison, we also included this minimal clinically important difference estimate.

Of the different statistical strategies that can be used to establish the minimal clinically important difference by the anchor-based method, we chose to use the receiver operating characteristics approach, which establishes the borderline between status 3 (somewhat improved) and status 4 (unchanged), maximising specificity and sensitivity. In order to establish the desirable clinically important difference, we used the receiver operating characteristics method to distinguish between status 2 (much improved) and status 3 (somewhat improved).

In order to calculate the standard error of measurement, we used data from an earlier publication,^12^ which established the correlation between two pre-operative VAS ratings of nasal obstruction in the same patients. The patients confirmed that their sense of nasal obstruction was the same at both ratings. This correlation factor was 0.84 for daytime and 0.85 for night-time.

### Statistical analyses

Descriptive statistics were expressed as numbers (percentages) and means (standard deviations (SDs)) for respondents’ demographics, nasal obstruction VAS score and status.

An analysis of variance with Scheffé post-hoc testing was used to compare the change in VAS scores for the five status groups. Spearman correlations were used to correlate status groups and the change in VAS scores.

All analyses were two-sided and statistical significance was defined as *p* < 0.05. Statistical analyses were performed using IBM SPSS® software (version 28.0).

We used the user-developed Stata command ‘cutpt’ to find the optimal cutpoint from a receiver operating characteristics curve with the Lui method that maximises the product of the sensitivity and specificity.

## Results

A total of 935 patients (664 males, 271 females), with a mean age of 37.7 years, had undergone septoplasty with or without turbinate surgery, between 2014 and 2019, and had completed both the pre- and post-operative questionnaires.

The pre- and post-operative VAS scores, the change in VAS scores after surgery and the global rating of the surgical result are presented in Table 1. The improvement in VAS scores after surgery was statistically significant both during the day and at night for the whole cohort and for all statuses except status 5 (worse).

**Table 1.** Pre-, post-operative and improvement nasal obstruction VAS scores for day and night*

	Pre-op		Post-op		Change		
Status^†^	*N*	VAS score (mean (SD))	*N*	VAS score (mean (SD))	*n*	VAS score (mean (SD))	*P*-values
Daytime							
– 1	140	64.1 (19.8)	140	4.7 (6.3)	140	59.4 (20.5)	<0.001
– 2	460	62.7 (20.5)	459	17.6 (13.2)	459	45.1 (22.9)	<0.001
– 3	242	60.7 (20.3)	242	41.5 (16.9)	242	19.2 (21.8)	<0.001
– 4	75	69.5 (19.8)	75	63.9 (17.4)	75	5.6 (18.1)	0.004
– 5	18	66.3 (23.0)	18	72.1 (21.1)	18	−5.8 (22.5)	0.287
– Total	935	63.0 (20.4)	934	26.6 (22.8)	934	36.6 (27.9)	<0.001
Night-time							
– 1	140	74.7 (18.0)	140	7.1 (8.8)	140	67.6 (19.0)	<0.001
– 2	460	74.5 (18.0)	455	24.4 (16.8)	455	50.0 (23.1)	<0.001
– 3	241	74.7 (17.8)	241	51.3 (18.8)	241	23.3 (19.2)	<0.001
– 4	75	80.9 (17.1)	74	74.6 (15.2)	74	7.1 (15.5)	<0.001
– 5	18	76.1 (17.1)	18	84.1 (13.1)	18	−8.0 (17.5)	0.063
– Total	935	75.1 (17.9)	928	33.9 (28.3)	928	41.2 (28.3)	<0.001

* According to global retrospective outcomes (status 1–5). ^†^Status 1 = completely improved, status 2 = much improved, status 3 = somewhat improved, status 4 = unchanged, and status 5 = worse. VAS = visual analogue scale; pre-op = pre-operative; post-op = post-operative; SD = standard deviation

Analysis of variance and post-hoc testing showed that the five global status groups had significantly different levels of improvement in VAS score (all *p* < 0.001), except that status 4 and 5 groups did not differ significantly from each other.

### Minimal clinically important difference

The anchor-based minimal clinically important difference estimates using the receiver operating characteristics approach are presented both in VAS scores and in the percentage change from baseline scores (Table 2).

**Table 2.** VAS scores and change from baseline of minimal clinically important difference, during day and night

Parameter	VAS (specificity / sensitivity)	% change from baseline
Daytime	9.5 (0.653 / 0.600)	15.1
Night-time	12.5 (0.722 / 0.689)	16.6

VAS = visual analogue scale

### Standard error of measurement

We calculated the standard error of measurement by establishing the SD of the measurements as the mean of the SDs of the pre- and post-operative day and night scores, respectively. We used the equation 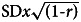, in which the correlation factor *r* (day = 0.84, night = 0.85) was from a former study.^12^ The standard error of measurement was 8.6 for daytime and 9.0 for night-time.

### Recall bias

The correlations between the change in VAS scores for obstruction after surgery and the global ratings (status 1–5) was 0.623 (*p* < 0.001) for daytime and 0.677 (*p* < 0.001) for night-time, which are well above the recommended threshold level of 0.37, suggesting minimal recall bias.^13^

### Gender

We also examined possible bias because of gender. No significant gender differences were found for any of the status groups (*p* = 0.526) or for change in day or night VAS ratings (*p* = 0.735).

### Age

Using a median split to evaluate the impact of age, patients aged 38–80 years showed significantly more improvement in both day and night VAS ratings of nasal obstruction than younger patients aged 17–37 years (both *p* < 0.005). However, this difference between young and old was only significant for status 1 (completely improved) and not for statuses 2–5. Therefore, age would not influence the calculation of the minimal clinically important difference, which involves statuses 3 and 4, nor that of the desirable clinically important difference using statuses 2 and 3.

### Desirable clinically important difference

Using receiver operating characteristics, we found the border between status 2 (much improved) and 3 (somewhat improved) to be a VAS change score of 28.5 (specificity = 0.791, sensitivity = 0.665) for daytime, and a VAS change score of 39.5 (specificity = 0.712, sensitivity = 0.797) for night-time. This correlates to a post-operative improvement of 45.2 per cent and 52.6 per cent respectively from baseline. The desirable clinically important difference was independent of gender and age.

## Discussion

We found that the mean change in VAS scores was statistically significant, even among patients who rated their global outcome as ‘unchanged’ (status 4). Thus, we found it important to establish a minimal clinically important difference that was clinically relevant. The anchor-based minimal clinically important difference value of obstruction for the whole cohort during the day was 9.5 and at night it was 12.5. This corresponds to an improvement in nasal obstruction of 15.1 per cent during the day and 16.6 per cent at night.

Several studies have assessed the clinically important improvement of nasal obstruction in relation to septoplasty. They have used different instruments and estimates, which makes comparisons between them difficult. We have, therefore, converted the nominal improvement scores of the different instruments to percentages of improvement from baseline.

In a study of 59 patients, Stewart *et al*.^1^ estimated the minimal clinically important difference using an anchor-based method. The authors found that Nasal Obstruction Symptom Evaluation survey scores (range, 0–100) showed a mean improvement from 67.5 to 23.1 after surgery. The improvement was compared to five-point Likert satisfaction scale findings, but very few patients reported a minimal change in breathing status. They did not specify their statistical approach for estimating the minimal clinically important difference. We used the receiver operating characteristics approach, and had an adequate number of patients reporting lower global ratings. We, therefore, believe that their minimal clinically important difference of 28.7 per cent from baseline, substantially higher than in our study, is less reliable.

Stewart *et al*.^1^ and Mondina *et al*.^2^ also calculated the minimal clinically important difference using the distribution-based method described by Guyatt *et al*.^14^ The minimal clinically important difference in the first study was found to be 3.9–5.9 Nasal Obstruction Symptom Evaluation survey points, representing a change of 5.8 per cent to 8.7 per cent, and in the second study, it was found to be 5–7.5 Nasal Obstruction Symptom Evaluation survey points, corresponding to a change of 8.6–13 per cent from baseline. Lodder and Leong^3^ used these minimal clinically important difference estimates to evaluate their results. These values are substantially lower than our anchor-based ones.

Other studies have established higher minimum levels of clinical improvement when evaluating nasal septoplasty outcome. Rhee *et al*.^5^ reviewed several studies reporting change in nasal obstruction ratings after nasal surgery. The average pre- and post-surgical scores were 65 and 23 points in studies using a Nasal Obstruction Symptom Evaluation survey (score range, 0–100) scale, 6.9 and –2.1, respectively, when using a VAS (score range, 0–10). As all studies showed a minimum improvement of 30 (Nasal Obstruction Symptom Evaluation survey) and 3 (VAS) points, they considered these levels to be clinically meaningful measures of success. These changes represent improvements of 46.2 per cent (Nasal Obstruction Symptom Evaluation survey) and 43.5 per cent (VAS).

Fuller *et al*.^6^ referred to the study by Rhee *et al*.,^5^ and used improvement of 46.2 per cent as a meaningful minimal clinically important difference estimate when assessing the usefulness of a quality of life instrument in quantifying the results of nasal surgery. As a secondary aim, we calculated the VAS score that distinguishes between the ‘somewhat’ and ‘much’ improved groups (statuses 2 and 3). These desirable clinically important differences were 28.5 for daytime and 39.5 for night-time, corresponding to improvements of 45.2 per cent and 52.6 per cent from baseline. We believe that their measures of success are comparable to our desirable clinically important difference.

Ziai and Bonaparte^7^ pre-operatively asked 67 patients who would later undergo septoplasty and turbinoplasty to quantify the degree of improvement in Nasal Obstruction Symptom Evaluation survey scores (range, 0–20) that they would define as a surgical success. The mean change in this rating was 5.3 from a baseline of 12.9 points, representing an improvement of 41.1 per cent, which is close to the mean values reported in the study by Rhee *et al*.^5^ and to our desirable clinically important difference.

Buckland *et al*.^4^ used the SNOT-22 subscale of obstruction (score range, 0–5) in 40 patients, and found an improvement from 3.9 to 1.3 points at three months after septal surgery. They defined improvement as a reduction of 1 point on this subscale, which would signify an improvement of 26 per cent (1:3.9). This is in between our minimal clinically important difference and desirable clinically important difference estimates. Singular definitions of improvement may make comparison with other studies difficult.

- Statistically significant improvements in nasal obstruction ratings after septoplasty are not necessarily clinically important
- The minimal clinically important difference in other organ-specific studies was calculated with a distribution-based method
- By instead using the recommended anchor-based method, the minimal clinically important difference was established at 15 per cent of the pre-operative score
- The minimal clinically important difference was not influenced by gender, age or recall bias
- Patients want better outcomes than the minimal clinically important difference
- Several studies have used higher clinical estimates as criteria for success; these were equivalent to our desirable clinically important difference, set at 45 per cent of the pre-operative value

A strength of our study is that it was conducted in a single hospital, with a large number of patients, using the same questionnaire pre- and post-operatively. The global and the VAS ratings were presented simultaneously in the same questionnaire and by mail only. As the purpose was to compare two instruments of improvement within subjects, confounding factors such as allergies, smoking, medication and quality of the surgery were eliminated.

In order to make our findings commensurable with those of other studies, we converted the results from nominal values to percentages of improvement. We realise that this is an indirect way of performing the comparisons, but we believe that it adequately expressed the commensurability of findings.

The anchor-based questionnaire method was used retrospectively. Global retrospective ratings seem to correlate better to the post-operative outcome than to the improvement scores. We found a good correlation between the change in VAS scores and global outcome ratings, so we believe that recall bias did not substantially influence the results.
